# Current Perspectives on ^89^Zr-PET Imaging

**DOI:** 10.3390/ijms21124309

**Published:** 2020-06-17

**Authors:** Joon-Kee Yoon, Bok-Nam Park, Eun-Kyoung Ryu, Young-Sil An, Su-Jin Lee

**Affiliations:** 1 Department of Nuclear Medicine & Molecular Imaging, Ajou University School of Medicine, Worldcup-ro 164, Suwon 16499, Korea; curies@hanmail.net (B.-N.P.); aysays77@naver.com (Y.-S.A.); suesj@naver.com (S.-J.L.); 2 Division of Magnetic Resonance, Korea Basic Science Institute, 162, Yeongudanji-ro, Cheongju 28119, Korea; ekryu@kbsi.re.kr

**Keywords:** positron emission tomography, ^89^Zr, monoclonal antibody, oncological imaging

## Abstract

^89^Zr is an emerging radionuclide that plays an essential role in immuno-positron emission tomography (PET) imaging. The long half-life of ^89^Zr (t_1/2_ = 3.3 days) is favorable for evaluating the in vivo distribution of monoclonal antibodies. Thus, the use of ^89^Zr is promising for monitoring antibody-based cancer therapies. Immuno-PET combines the sensitivity of PET with the specificity of antibodies. A number of studies have been conducted to investigate the feasibility of ^89^Zr immuno-PET imaging for predicting the efficacy of radioimmunotherapy and antibody therapies, imaging target expression, detecting target-expressing tumors, and the monitoring of anti-cancer chemotherapies. In this review, we summarize the current status of PET imaging using ^89^Zr in both preclinical and clinical studies by highlighting the use of immuno-PET for the targets of high clinical relevance. We also present ^89^Zr-PET applications other than immuno-PET, such as nanoparticle imaging and cell tracking. Finally, we discuss the limitations and the ongoing research being performed to overcome the remaining hurdles.

## 1. Introduction

Positron emission tomography (PET) is a widely used imaging technology in clinical oncology. Among the radiotracers used for PET imaging, ^18^F-fluorodeoxyglucose (FDG) has played a remarkable role in staging, restaging, detecting recurrences, and predicting the prognosis of various cancers [1]. Although ^18^F-FDG is still a key radiotracer, recently, radiopharmaceuticals other than ^18^F-FDG have been thoroughly investigated to predict and monitor therapeutic responses along with the development of targeted therapies [2]. Radioisotopes with short half-lives, such as ^18^F (t_1/2_ = 110 min), ^11^C (t_1/2_ = 20 min) and ^13^N (t_1/2_ = 10 min), which are common in clinical practice, have the advantage of low radiation exposure. However, they are not optimal for long circulating probes, such as the monoclonal antibody (mAb). Therefore, radiolabeling with long-lived radioisotopes such as ^124^I (t_1/2_ = 4.2 days), ^64^Cu (t_1/2_ = 12.7 h), and ^89^Zr (t_1/2_ = 3.3 days) is required for the better assessment of the biodistribution of such tracers [3,4].

**Figure 1:**
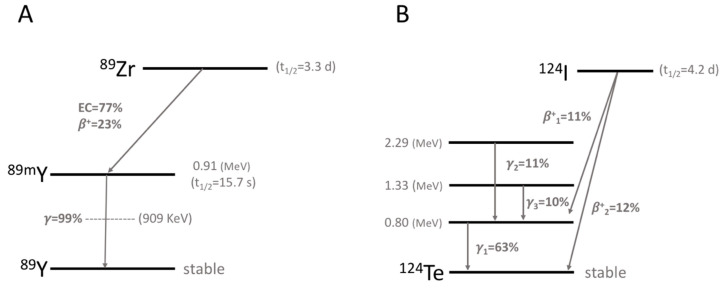
Radioactive decay scheme for ^89^Zr (**A**) and ^124^I (**B**).

^89^Zr is a positron-emitting radionuclide that can be produced by a medical cyclotron. The first production of ^89^Zr for the labeling of mAb was performed in 1986 by proton bombardment using a solid target, ^89^Y(p,n)^89^Zr [5]. ^89^Zr decays in two ways (positron emission, 23% and electron capture, 77%) by emitting two important γ-rays: 909 KeV photons during the deactivation of ^89m^Y and 511 KeV photons from the positron–electron annihilation (Figure 1A). These photons can be separated by setting the energy windows of PET. In addition, they do not coincide because of the long half-life of ^89m^Y. ^89^Zr has a relatively short positron range by emitting low energy β^+^ rays (E_β+,ave_ = 396 KeV), which facilitates high-resolution PET imaging.

When ^89^Zr is used for immuno-PET imaging, it has a few advantages over another long-life positron emitter, ^124^I. As the positron range of ^89^Zr is shorter than that of ^124^I due to its lower positron energy (E_β+,ave_ for ^124^I = 819 KeV, Figure 1B), ^89^Zr-PET has a superior spatial resolution to ^124^I-PET [6,7]. ^124^I does not residualize (trapped within the cells after catabolism of the radiolabeled mAbs) and is rapidly released from the cells when it is labeled to mAbs. Meanwhile, ^89^Zr internalizes and residualizes after binding to the surface of cells. This difference results in 1.5- to 3-fold higher tumor uptake for ^89^Zr-labeled mAb than for ^124^I-labeled mAb [7,8]. Some disadvantages of ^124^I are its high cost, high impurity, and long production time. ^89^Zr can be produced at a low cost within a few hours and is easy to purify because fewer contaminants must be removed.

**Figure 2:**
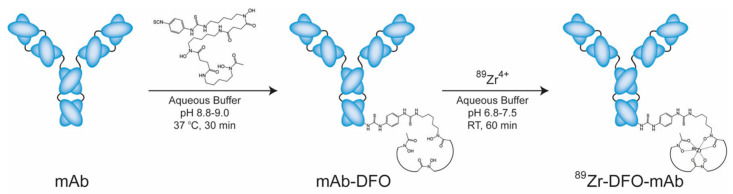
Scheme of the bioconjugation and radiolabeling of ^89^Zr-desferrioxamine B (DFO)-J591. This is adapted from Zeglis, B. M., Lewis, J. S. The bioconjugation and radiosynthesis of ^89^Zr-DFO-labeled antibodies. *J. Vis. Exp.* **2015**, *96*, e52521, doi:10.3791/52521.

As ^89^Zr is a metallo-radionuclide, it is stably bound as long as its bifunctional chelator is conjugated to its probes. Since it was first evaluated in 1992, desferrioxamine B (DFO) has been the most popular chelator for ^89^Zr labeling (Figure 2) [9]. DFO originated from the iron-binding siderophores and consists of hydroxamate groups as the binding site for ^89^Zr [10]. With the successful labeling of ^89^Zr to mAbs using DFO, various ^89^Zr-chelating ligands have been developed [11].

## 2. ^89^Zr-PET Imaging in the Literature

With the success of synthesizing ^89^Zr-labeled antibodies, the number of preclinical and clinical studies related to ^89^Zr-PET imaging has markedly grown over the last three decades. As of early 2019, more than 300 original articles on the production, radiolabeling chemistry, and preclinical and clinical studies of ^89^Zr have been published according to a search of Pubmed. When classified by the tracers labeled, antibodies (whole or fragments) and antibody mimetics, occupy more than 70% of those studies, followed by nanoparticles (NPs), proteins, peptides, and cells. For the last 10 years, the number of antibodies and antibody fragments approved by the Food and Drug Administration (FDA) has greatly increased from 22 (2010) to 93 (2018). Among these, 17 antibodies were labeled to ^89^Zr and evaluated as PET imaging agents (Table 1). Trastuzumab [12,13,14,15,16] is the most frequently studied antibody, followed by bevacizumab [17,18,19,20,21], cetuximab [22,23,24], and rituximab [25,26]. Consequently, human epidermal growth factor receptor-2 (HER2), epidermal growth factor receptor (EGFR), vascular endothelial growth factor-A (VEGF-A), cluster of differentiation (CD) 20, and prostate specific membrane antigen (PSMA) are the most frequently explored targets. Researchers have also evaluated several investigational drugs, such as imgatuzumab [27]. Several clinical studies have already been published, but preclinical studies are still much more common than in clinical studies.

The first small animal PET images using ^89^Zr were reported in 1997 [28]. ^89^Zr was labeled to mAb 323/A3, which was derived by immunizing mice with human breast cancer cells. PET images were acquired with a clinical PET camera 55 h after intravenous injection and showed specific tumor uptake in mice bearing ovarian cancer xenografts. The first human study was published in 2006 [29]. In patients with squamous head and neck cancers, PET imaging with ^89^Zr-labeled chimeric mAb U36 localized cervical lymph node metastasis with a high accuracy (93%). ^89^Zr immuno-PET images were obtainable for up to 144 h after injection.

The term ‘immuno-PET’ first appeared in the literature in 1999 [30]. By combining the sensitivity of PET imaging and the specificity of antibodies, immuno-PET imaging has become a promising tool for monitoring the heterogeneity of specific gene expression and predicting the efficacy of targeted therapies. From a technical perspective, immuno-PET is basically an extension of two nuclear medicine modalities, radioimmunoscintigraphy and radioimmunotherapy. Immuno-PET images have a better resolution than the radioimmunoscintigraphies obtained by gamma cameras; in addition, immuno-PET images can be used as a surrogate marker for radioimmunotherapy.

In this review, we summarize the current status of ^89^Zr-PET imaging by highlighting the application of immuno-PET for the targets of high clinical relevance. For every topic, oncological applications will primarily be addressed. We also introduce ^89^Zr-PET imaging other than immuno-PET, such as nanoparticle imaging. Finally, the limitations and current studies to overcome the hurdles are discussed.

## 3. Preclinical and Human Studies in ^89^Zr Immuno-PET Imaging

### 3.1. Immuno-PET Targeting HER2

HER2 belongs to a member of the ErbB family receptor tyrosine kinases: EFGR, HER2, HER3, and HER4. HER2 plays a critical role in the angiogenesis, differentiation, metastasis, proliferation, and survival of cancer cells. The increased expression of HER2 is found in a number of cancers, including breast cancer, colon cancer, lung cancer, ovarian cancer, and stomach cancer [31]. Trastuzumab is one of the most effective therapeutic mAbs and also occupies the majority of HER2-targeting immuno-PET studies. On the other hand, only a few studies explored the feasibility of ^89^Zr-pertuzumab PET imaging [32,33].

Gamma camera imaging with ^111^In (t_1/2_ = 2.8 days) was used for years as a surrogate marker for the optimal distribution of therapeutic mAbs [34,35]. ^89^Zr-trastuzumab is specific for HER2 positive tumors and shows a similar biodistribution to ^111^In-trastuzumab when injected into mice bearing HER2-positive tumors [12]. The only difference is a slight increase in the bone marrow uptake for ^89^Zr-trastuzumab, which was probably due to the accumulation of free ^89^Zr. Considering the advantage of more accurately quantifying the tracer uptake and dose calculation of PET, ^89^Zr-trastuzumab PET imaging could be a better option for monitoring therapeutic mAbs.

The optimal time interval between the intravenous injection of ^89^Zr-trastuzumab and PET imaging for a high tumor to background contrast is 4–8 days [13,16,33,36]. If only an imaging study is performed, the optimal dose for ^89^Zr-trastuzumab is 50 mg. Meanwhile, the optimal dose reduces to 10 mg during trastuzumab therapy because the hepatic clearance of ^89^Zr-trastuzumab is slow due to the high blood concentration of trastuzumab and thus a smaller dose can be used to achieve good tumor uptake [16]. ^89^Zr-trastuzumab can detect most metastatic sites (liver and bone) in patients with HER2-positive breast cancer [16]. On the other hand, it was shown to detect unsuspected HER2-positive metastatic sites even in patients with HER2-negative primary breast cancer, indicating the heterogeneity of the tumors [14,37]. Evaluating the HER2 status of tumors is crucial for clinical decision making for patients planning anti-HER2 therapy. A whole body evaluation of HER2 expression with ^89^Zr-trastuzumab PET is helpful, particularly when a tumor biopsy is unfeasible. The imaging results for HER2 expression may support or change the treatment plans [38,39].

The responses to anti-HER2 therapy other than mAbs can be monitored by ^89^Zr-trastuzumab PET. The heat shock protein 90 inhibitor-induced early downregulation and the late recovery of HER2 expression was shown via a ^89^Zr-trastuzumab PET in xenograft models [40,41]. The ^89^Zr-trastuzumab PET also displayed a tyrosine kinase inhibitor (afatinib and lapatinib)-induced reduction in HER2 expression [15,42]. The heterogeneous uptake of ^89^Zr-trastuzumab under PET reflects the genomic heterogeneity of tumors, which is related to the mixed response to afatinib treatment [43].

Based on a few human studies, the estimated radiation dose of ^89^Zr-trastuzumab PET for patients is about 0.54 mSv/MBq [13,16]. Considering the usual dose (37–74 MBq) for ^89^Zr-trastuzumab PET, this value is 2–4 times higher than that of ^18^F-FDG PET. This is one of the major pitfalls to overcome before adopting ^89^Zr-PET for clinical use.

Similar to ^89^Zr-trastuzumab, the optimal time for ^89^Zr-pertuzumab PET is 5–8 days after injection. ^89^Zr-pertuzumab was able to detect HER2-positive brain metastasis in a patient with heterogeneous HER2 expression for primary tumors, indicating the usefulness of immuno-PET imaging for predicting the response to targeted therapy [33]. ^89^Zr-pertuzumab was safe; however, the effective dose was as high as that of ^89^Zr-trastuzumab (0.54 mSv/MBq).

Next-generation antibodies include antibody–drug conjugates (ADCs), immune checkpoint inhibitors, mAbs fused with biological toxins or cytokines, and fragmented antibodies. Immuno-PET could be helpful in the development of ADCs by identifying the presence of a certain biomarker and predicting the therapeutic response (“companion diagnostics”) [44]. Trastuzumab emtansine (T-DM1) is a good example of an ADC that covalently links a cytotoxic agent, DM1, to mAb. For progressive triple-negative breast cancers, baseline ^89^Zr-trastuzumab PET was able to predict the treatment failure of T-DM1. The early response evaluated by PET after 1 cycle of T-DM1 treatment correlated well with the prognosis [45].

### 3.2. Immuno-PET Targeting EGFR

EGFR, also known as HER1, is also a member of the ErbB family receptor tyrosine kinase, and its overexpression of EGFR can be observed in breast cancer, head-and-neck cancer, non-small cell lung cancer, renal cancer, ovarian cancer, and colon cancer. EGFR is involved in the differentiation, proliferation, and survival of various cancer cells and is thus related to a poor prognosis, shorter survival, aggressive growth, and invasiveness of cancers [46].

^177^Lu (t_1/2_ = 6.7 days; β, 0.497 MeV) is a β^−^emitting radionuclide, which is promising for cancer therapy when it is labeled to antibodies or peptides [40,47]. To improve therapeutic efficacy, it is essential to predict its in vivo distribution. ^89^Zr-cetuximab and ^177^Lu-cetuximab show similar biodistribution over organs, except for a higher bone marrow uptake for ^89^Zr-cetuximab at 48, 72 and 144 h after injection [48]. With careful consideration of the bone marrow uptake, ^89^Zr-PET may be useful for selecting candidates for radioimmunotherapy or radionuclide peptide therapy. 

EGFR-expressing malignant tumors that are refractory under conventional chemotherapy and radiotherapy may benefit from targeted therapy with cetuximab. However, the tumoral heterogeneity of EGFR expression in some patients hinders successful treatment by cetuximab. PET imaging with ^89^Zr-cetuximab presented a difference in the EGFR expression in patients with head-and-neck cancers and in those with advanced colorectal cancers, as revealed by the standardized uptake value (SUV) [22,23]. For colorectal cancers, the tumor uptake of ^89^Zr-cetuximab correlated well with the therapeutic outcome with cetuximab [23]. The uptake of ^89^Zr-nimotuzumab and ^89^Zr-panitumumab was also specific and correlated well with the EGFR expression of tumors in colorectal and breast cancer models [49,50].

The whole-body effective dose of ^89^Zr-cetuximab PET (0.61 mSv/MBq) was similar to that of ^89^Zr-trastuzumab (0.54 mSv/MBq). The liver has the highest absorbed dose (2.60 mGy/MBq), which is 0.51 mGy/MBq for red marrow [24]. On the other hand, ^89^Zr-panitumumab has a much lower whole-body effective dose at 0.26 mSv/MBq. Additionally, PET images are obtainable after 5–7 days by injecting as little as 37 MBq of ^89^Zr-panitumumab [51]. Considering the radiation exposure, ^89^Zr-panitumumab is a safer radiopharmaceutical than ^89^Zr-cetuximab.

Various fragmented antibodies and antibody mimetics have been developed to overcome the shortcomings of whole antibodies. Affibody is an engineered protein that consists of 59 amino acid (~6 kDa). Rapid tumor penetration and blood clearance are advantages of the affibody. The ^89^Zr-labeled EGFR-specific affibody, ZEGFR:03115, showed tumor uptake as early as 3 h after injection. There was a good correlation between the tumor uptake of the ^89^Zr-affibody and EGFR expression [52]. These findings demonstrate that ^89^Zr-immuno PET imaging can be a good predictor for targeted antibody therapy.

### 3.3. Immuno-PET Targeting VEGF-A

VEGF-A binds to VEGF receptors 1 and 2 and then acts as a proangiogenic factor in normal tissues and tumors. VEGF is involved in endothelial cell proliferation, survival, migration, and vascular permeability. The overexpression of VEGF is observed in many types of cancers [53].

Bevacizumab binds to all isoforms of VEGF-A and is the only FDA-approved mAb used for immuno-PET imaging. ^89^Zr-bevacizumab PET has a high sensitivity (96%) for detecting VEGF expressing primary breast cancers and high specificity (100%) for lymph node metastasis [21]. The ^89^Zr-bevacizumab uptake given by the maximum SUV is correlated with the VEGF-A concentration and proliferation index of the primary tumors.

Unlike ^18^F-FDG PET, ^89^Zr-bevacizumab PET shows a very low brain uptake, enabling the visualization of VEGF-A expressing tumors in the brain and adjacent organs [18,54]. The von Hippel–Lindau (VHL) syndrome is a rare genetic disorder characterized by a variety of tumors including hemangioblastomas, retinal angiomas, and visceral cysts. Locally produced VEGF-A mediates angiogenesis during disease manifestation; whole-body surveillance for VEGF expressing tumors is required for anti-angiogenesis therapy with bevacizumab [18]. Diffuse pontine glioma is a rare brainstem tumor that is refractory to systemic chemotherapies. VEGF is overexpressed in diffuse pontine glioma. Thus, bevacizumab therapy can be effective in an individual patient [54]. Immuno-PET using ^89^Zr-bevacizumab may be helpful in identifying potential responders to treatment among these patients.

^89^Zr-bevacizumab PET can be used for monitoring anti-angiogenesis treatment. ^89^Zr-bevacizumab uptake, VEGF-A concentration, and microvessel density in ovarian cancer xenografts decreased through the treatment of a mammalian target of the rapamycin inhibitor, everolimus [55]. In patients with metastatic renal cell carcinoma, the change in the maximum SUV between 2 and 6 weeks was an indicator for the response of the everolimus treatment. PET-guided treatment led to stable disease for all patients [56]. The therapeutic response to bevacizumab/interferon-α can be assessed by interim ^89^Zr-bevacizumab PET. Only half of the lesions had a ^89^Zr-bevacizumab uptake because of the heterogeneous expression of VEGF [20]. Sunitinib, a multi-targeted tyrosine kinase inhibitor, exerts an anti-angiogenic effect by inhibiting VEGF receptors 1 and 2 [57]. Immuno-PET visualized the rebound growth of cancers via the discontinuation of sunitinib [20,58]. The ^89^Zr-bevacizumab uptake in metastatic renal cell carcinomas decreased by 14.3% after 2 weeks of therapy but increased by 72.6% at 2 weeks after discontinuation [20]. ^89^Zr-ranibizumab PET also visualized the rebound of ovarian cancer xenografts after the discontinuation of sunitinib treatment [58].

Apart from oncologic diseases, ^89^Zr-bevacizumab PET may be used for detecting vulnerable plaques. VEGF release in atherosclerotic plaques is known to make the plaques vulnerable. The ^89^Zr-bevacizumab PET detected all the carotid endarterectomy specimens ex vivo and correlated with the VEGF expression revealed by immunohistochemical staining [19].

### 3.4. Immuno-PET Targeting CD20

CD20 is a non-glycosylated phosphoprotein that is expressed on the surface of B-cell hematopoietic malignancies such as B-cell non-Hodgkin lymphoma (NHL) and chronic lymphocytic leukemia [59]. Various CD20-targeting mAbs are applied to treat B-cell-related malignancies and autoimmune diseases. To date, ^89^Zr-PET imaging with five different mAbs has been applied: ibritumomab, rituximab, tositumomab, obinutuzumab, and ofatumumab.

The first FDA-approved radioimmunotherapy was ^90^Y-ibritumomab tiuxetan (Zevalin^®^, Acrotech Biopharma, LLC, East Windsor, NJ, USA). As ^90^Y is a pure β-emitting radioisotope, ^111^In has been used as a surrogate to predict the response to ^90^Y [60]. ^89^Zr is a candidate to replace ^111^In because ^89^Zr and ^90^Y (t_1/2_ = 2.7 days) have similar physical half-lives. The biodistribution of ^89^Zr-zevalin was not affected by co-injected ^90^Y-zevalin, resulting in a similar biodistribution between ^89^Zr-zevalin and ^90^Y-zevalin, except for liver and bone marrow [61,62]. ^89^Zr-zevalin showed a significantly higher uptake in the liver, thigh bone and sternum at 72 and 144 h after injection, as compared to ^90^Y-zevalin [61]. In patients with NHL, pre-treatment ^89^Zr-zevalin PET imaging can be used to determine the dose-limiting organs in ^90^Y-zevalin treatment [62].

^89^Zr-rituximab PET is useful for detecting CD20 expression in patients with diffuse large B-cell lymphoma. The tumor uptake of ^89^Zr-rituximab showed a high concordance with CD20 expression when studied with immunohistochemistry [25]. ^89^Zr-rituximab PET also helped prevent invasive nerve biopsy while determining an optimal therapy for neurolymphomatosis [63].

Besides hematologic malignancies, patients with rheumatoid arthritis may benefit from ^89^Zr-rituximab PET. B cell-depletion therapy with rituximab is based on the role of the B lymphocyte in the pathogenesis of rheumatoid arthritis and appears to be efficient in some patients with refractory disease [64]. The ^89^Zr-rituximab uptake of hand joints in PET was significantly higher in the rituximab-responder group. This uptake correlated inversely with the B-cell count in the lymph nodes after treatment [26]. A ^89^Zr-rituximab PET may thus be helpful in selecting patients who will be responsive to rituximab therapy.

### 3.5. Immuno-PET Targeting PSMA

PSMA is a transmembrane protein that is heterogeneously expressed on prostate epithelial cells. PSMA is highly expressed in prostate cancers and correlates with the metastasis and progression of prostate cancer [65]. Two important mAbs, 7E11 and J591, are associated with ^89^Zr immuno-PET imaging. 7E11 is already used for immunoscintigraphy in the form of ^111^In-labeled capromab pendetide (ProstaScint^®^, Cytogen Corporation, Princeton, NJ, USA). ^89^Zr-7E11 PET has been infrequently studied because 7E11 binds to the intracellular domain of PSMA [66]. On the other hand, J591 binds to the extracellular domain of PSMA. Thus, most immuno-PET studies are associated with J591 mAb and its minibody (IAB2M).

^89^Zr-J591 PET is used to detect PSMA-expressing tumors [67,68,69]. Early studies with ^89^Zr-J591 PET have shown inconsistent results for the diagnostic performance of primary prostate cancers [67,68]. For prostate cancer without metastasis (n = 11), there was no difference in Gleason’s score between ^89^Zr-J591 (+) and ^89^Zr-J591 (−) tumors [67]. Meanwhile, for those with metastatic prostate cancers (n = 10), the sensitivity for detecting primary tumors increased to 100%. A ^89^Zr-J591 PET is also useful for detecting bone metastasis compared to conventional imaging modalities [68]. A subsequent study with a larger population (n = 50) of castration-resistant prostate cancers suggested that ^89^Zr-J591 PET had a higher sensitivity for bone metastasis than conventional imaging methods, while conventional imaging methods were more sensitive for soft tissue lesions [69]. Based on these results, ^89^Zr-J591 PET could be used for detecting bone metastasis in advanced prostate cancers.

^89^Zr-J591 PET guided biopsy has very high accuracy for prostate cancer [70]. Compared to ^18^F-FDG, the longer half-life of ^89^Zr can help avoid reinjection for biopsy guidance.

^89^Zr-labeled IAB2M showed a rapid accumulation in tumors and a fast clearance from the blood within 24 h in a prostate cancer model [71]. The ^89^Zr-IAB2M uptake of bone and lymph node metastases was discernible in as little as 24 h and lasted up to 120 h in patients with prostate cancer [72]. The ^89^Zr-IAB2M PET clearly visualized recurrent cerebral glioma and metastatic brain tumors from lung cancer, and the tumor to background contrast of the ^89^Zr-IAB2M uptake was shown to be higher than that of ^11^C-methionine PET [73]. The ^89^Zr-IAB2M uptake correlated with PSMA expression [73].

The calculated effective dose from the ^89^Zr-J591 PET is 0.38 mSv/MBq (14.1 mSv/mCi) [68]. The radiation dose from 37–74 MBq ^89^Zr-J591 is slightly higher than that of 370 MBq ^18^F-FDG. The effective dose from the ^89^Zr-IAB2M PET is 0.41–0.68 mSv/MBq [72,74]. The radiation exposure to medical staff and family is lower than 1 mSv for the first week [72].

### 3.6. Immuno-PET Targeting CD44

CD44 is a cell-surface glycoprotein involved in many biological processes. The v6 splice variant of CD44 (CD44v6) is involved in tumorigenesis, tumor cell invasion, and metastasis. CD44v6 is expressed in various cancers, predominantly in squamous cell carcinomas [75].

^89^Zr-labeled mAbs against CD44v6 may be used for the detection of tumors and the prediction of therapies [29,76,77]. ^89^Zr-labeled chimeric mAb, U36, showed a similar biodistribution to its ^90^Y-labeled therapeutic counterpart, which enables it to predict the response of radioimmunotherapy [76]. In head-and-neck cancer patients, the diagnostic performance of ^89^Zr-U36 was comparable to that of conventional imaging methods (29). The ^89^Zr-labeled humanized mAb, RG7356, also showed specific tumor uptake in CD44+ tumors. The uptake of ^89^Zr-RG7356 correlated with CD44 expression [77].

### 3.7. Immuno-PET Targeting HER3

HER3 is also a member of the ErbB family of receptor tyrosine kinases. Due to the lack of intrinsic kinase activity, HER3 forms active heterodimers with other members of the ErbB family and mediates the proliferation, invasion, and metastasis of cancers and resistance to chemotherapy [78].

HER3 expression on various tumors has also been visualized, and the treatment response by ^89^Zr-anti-HER3 PET has been predicted. PET using ^89^Zr-labeled lumretuzumab or GSK2849330 successfully demonstrated specific tumor uptake in patients with HER3-expressing tumors [79,80]. It is known that HER3 upregulation during anti-HER therapy is associated with resistance. The tumor uptake of the ^89^Zr-labeled anti-HER3 affibody, ZHER3:8698, increased under AUY922 treatment and correlated with the HER3 upregulation in MCF-7 xenografts [81].

### 3.8. Immuno-PET Using Immune Checkpoint Inhibitors

Recently, immunotherapies using immune-checkpoint inhibitors have drawn clinical attention and have been actively investigated for patients who have tumors that are refractory to conventional chemotherapies. The programed death ligand 1 (PD-L1) of tumor cells binds to the programed cell death protein 1 (PD-1) of T-cells, resulting in a suppression of the killing effect of T-cells [82]. Various mAbs have been discovered to act against PD-L1 (atezolizumab, avelumab, and durvalumab) and PD-1 (pembrolizumab, nivolumab, and cemiplimab). Of these, atezolizumab, pembrolizumab, and nivolumab have been developed as potential immuno-PET imaging agents. In an animal model, ^89^Zr-nivolumab PET demonstrated PD-1 expression in T-cells that were activated by human peripheral blood lymphocyte engraftment [83]. In cancer patients, ^89^Zr-atezolizumab distributes to tumors and various organs, including normal lymphoid tissues. The ^89^Zr-atezolizumab uptake correlated with overall survival and progression free survival [84]. However, most studies are currently preclinical; thus, further clinical studies are required to corroborate the usefulness of ^89^Zr-atezolizumab.

### 3.9. Immuno-PET Targeting Lymphocyste Markers

Immunotherapies for refractory cancers, by increasing tumoral immune response, have shown durable effects in some patients who are responsive. The detection of T-lymphocyte infiltration into the tumor microenvironment is crucial for the early prediction of treatment efficacy [85]. Whole mAbs or engineered antibodies against CD3, CD4 and CD8 have been explored to monitor T-lymphocytes. The ^89^Zr-anti-CD3 showed a high tumoral uptake and a low background in mice bearing syngeneic tumors. The tumor-to-blood ratio of ^89^Zr-anti-CD3 was 11.5-fold higher than that of the isotype control [86]. The tumoral uptake of ^89^Zr-anti-CD3 showed a good correlation with the response to anti-cytotoxic T-lymphocyte antigen-4 therapy in a murine xenograft model. The tumor volume of the high uptake group was significantly smaller than that of the control or the low uptake group [87]. Moreover, ^89^Zr-anti-CD3 did not change total T-lymphocyte count, but induced a decrease in the CD4/CD8 ratio [86]. CD4 and CD8 T-lymphocytes can be detected by immuno-PET using ^89^Zr-labeled cys-diabodies against CD4 and CD8. Serial PET imaging at 2, 4 and 8 weeks after hematopoietic stem cell therapy demonstrated the repopulation of T-lymphocytes in lymph nodes and spleen [88]. PET imaging using a recently developed bispecific antibody targeting both T-lymphocytes and tumor cells also allows to detect tumor-infiltrating lymphocytes [89].

## 4. ^89^Zr-PET Imaging Other than Immuno-PET

### 4.1. ^89^Zr-Labeled Nanoparticles PET

Various radionuclides including ^198^Au, ^111^In, ^64^Cu, ^125m^Te, ^188^Re, ^166^Ho, and ^99m^Tc have been used for nanoparticle-based nuclear medicine imaging and therapy [90]. Dozens of studies concerning ^89^Zr-labeled NPs have already been reported, although only a few are clinical. Researchers suggest that ^89^Zr-labeled NPs (liposomal NPs, nanocolloids, mesoporous silica NPs, dextran NPs, chitosan NPs, etc.) are also promising for tumor detection, the development of nanoparticle drugs, the monitoring of drug delivery, inflammation imaging, and tumor-associated macrophage (TAM) imaging.

TAMs lead to disease progression in cancer cells by modulating the tumor microenvironment and are thus potential targets for anti-cancer therapy. To predict the efficacy of anti-TAM therapy, it is crucial to monitor the quantity and distribution of TAMs. ^89^Zr-labeled natural high-density lipoprotein (HDL) and dextran NPs showed favorable tumor uptake [91,92]. The co-localization of these radiotracers with a macrophage was revealed by histology and fluorescent imaging [91,92]. These results suggest that ^89^Zr-labeled NPs can be a good tool for monitoring anti-TAM therapy.

Several studies presented evidence for ^89^Zr-labeled nanocolloidal albumin as a PET imaging agent for sentinel lymph mapping [93,94,95,96]. In patients with early colon cancers or oral cavity cancers, PET detected sentinel lymph nodes with a very high sensitivity [95,96]. Due to the long half-life of ^89^Zr, sentinel lymph node mapping using ^89^Zr-NPs has the advantages of accomplishing both PET imaging and intraoperative probe detection via the single injection of a radiotracer, even though surgical procedures are performed on another day.

^89^Zr-labeled NPs for atherosclerotic plaques are good examples of inflammation imaging. HDL mimetic infusion has been studied for years as a method to reduce cardiovascular risk. One of the reasons for the failure of HDL mimetic infusion is its low target delivery. ^89^Zr-labeled natural HDLs and HDL mimetics are delivered to atherosclerotic plaques by being trapped in the macrophages [97,98]. The uptake of ^89^Zr-labeled HDL mimetics, CER-001, was slightly higher in plaques than in non-plaque walls and correlated well with the contrast enhancement by magnetic resonance imaging (98). Recent animal studies revealed that ^89^Zr-labeled dextran or hyaluronan NPs have the ability to detect atherosclerotic plaques and monitor anti-inflammatory therapy [99,100].

### 4.2. ^89^Zr-Induced Cerenkov Luminescence Imaging and Therapy

The Cerenkov effect was characterized by Pavel A. Cerenkov in 1934 as the radiation emitted when charged particles (β^+^, β^−^, α) travel through an optically transparent insulating material with a velocity that exceeds the speed of light. Cerenkov luminescence imaging has been exploited in a number of preclinical studies. The β^+^ particles emitted by ^89^Zr also produce Cerenkov luminescence. Using ^89^Zr-J591 and luminescence imaging, prostate cancers were visualized in animal models [101].

Photodynamic therapy requires external light to activate the photosensitizers for cancer therapy. Cerenkov radiation from ^89^Zr can be used as a light source for this purpose. ^89^Zr-labeled mesoporous silica NPs have ^89^Zr and photosensitizers inside their hollows. In a breast cancer model, the tumor suppression effect was greater for the ^89^Zr-labeled mesoporous silica NPs than for the NPs only, or for the control [102]. The advantage of ^89^Zr over conventional approaches is that ^89^Zr’s long half-life facilitates long-term photodynamic therapy.

### 4.3. Cell Tracking with ^89^Zr

Due to its old modality, radiolabeled leukocytes, cell tracking is nothing new for nuclear medicine imaging. Thus, it has already been adopted for various types of cell tracking. Using nuclear medicine imaging to evaluate the early distribution and viability of radiolabeled stem cells is a notable example [103]. With the development of cancer immunotherapy, tracking therapeutic cells is becoming more important for predicting the effectiveness of a therapy. ^89^Zr has a favorable physical half-life for tracking cells in vivo. Additionally, similar to ^111^In-oxine, ^89^Zr-oxine can be labeled to cells directly.

Chimeric antigen receptor (CAR) T-cells are transduced to locate specific targets on the surface of tumors. A few drawbacks of CAR T-cells include their poor tumor-targeting ability and normal tissue toxicity [104]. The prediction of therapeutic efficacy by cell tracking is critical to overcome these shortcomings. Direct labeling with ^89^Zr-oxine allowed the visualization of CAR T-cell migration to tumors in a glioblastoma model [105]. Labeling with ^89^Zr-oxine did not affect the viability and function of cells. The fragmented antibody F(ab’)_2_ for T-cell receptors is another candidate that showed high sensitivity for T-cells in an animal model. Transduced cells as small as 4.7 × 10^4^ were detected via PET imaging, and the tumor uptake quantity was proportional to the number of injected cells [106].

^89^Zr-desferrioxamine-N-chlorosuccinimide (DBN) is also actively studied as a direct cell labeling method. Unlike ^89^Zr-oxine, ^89^Zr-DBN binds covalently to the amine groups of cell surface membrane proteins [107]. Although the labeling efficiency was low to moderate (30~50%), ^89^Zr-DBN was stably bound to human mesenchymal stem cells (hMSCs) for up to 7 days without deteriorating the cellular viability [107]. PET imaging using ^89^Zr-hMSC that were delivered to the adventitia of the outflow vein of arteriovenous fistula allowed to track transplanted hMSCs for 3 weeks [108]. More than 90% of the transplanted cells were detected at the site of delivery on day 4, which was decreased by 20% on day 21 [108]. ^89^Zr-DBN was also labeled to hepatocytes with a labeling efficiency of 20% [109]. The initial amount of homing cells and the subsequent retention was monitored up to 48 h by PET imaging [109].

## 5. Discussion and Summary

Immuno-PET using ^89^Zr has the advantage of a high resolution and high specificity. ^89^Zr immuno-PET visualizes the expression of a variety of genes and reflects the status of tumor heterogeneity. Therefore, ^89^Zr immuno-PET is a useful modality to predict and evaluate the response of therapeutic mAbs and that of some targeted therapies.

A few points in previous studies should be carefully considered before clinical use. First, as the production of ^89^Zr requires a medium-energy cyclotron and a solid target system, the use of ^89^Zr is currently restricted to a small number of countries and institutes. Recent improvements in the yttrium target design facilitated the production of a clinical amount of ^89^Zr, which may provide a solution to this shortage in supply [110,111]. Another option for the production of ^89^Zr is using a liquid target. Irradiating the aqueous solution of yttrium nitrate (Y(NO_3_)_3_ 6H_2_O) generated a significant amount of ^89^Zr (0.27 ± 0.05 GBq/μA)—enough for clinical use in a single institute [112]. A second-generation solution target was employed to reduce the problems of in-target salt precipitation and unstable target pressure. A new target led to double the production quantity of ^89^Zr (~370 MBq in 2 h), which is sufficient for a small number of patients [113].

Second, as shown in previous studies, a significant amount of ^89^Zr accumulates in the bone marrow when entered into the circulatory system. As most dechelated forms of ^89^Zr (^89^Zr-chloride, ^89^Zr-citrate, and ^89^Zr-oxalate) accumulate in the bone marrow, this process is attributed to the instability of ^89^Zr-DFO chelation [30]. Recent studies in radiochemistry are paving the way toward improving stability by changing the structure of DFO or exploiting new chelators [114,115,116,117,118,119,120]. A decrease in bone marrow uptake is good for increasing sensitivity to lesions, as well as decreasing bone marrow toxicity for patients.

Compared to ^18^F-FDG and conventional computed tomography, patients generally receive higher radiation from ^89^Zr-labeled mAb PET—approximately 20–40 mSv for 37–74 MBq ^89^Zr. To reduce the radiation dose, a groundbreaking improvement in PET technology is necessary. Digital PET is a good example of successful dose reduction due to the enhanced sensitivity from using a PET equipped with digital silicon photomultipliers [121]. The high sensitivity of digital PET makes it possible to acquire images either with a shorter scan time or with a lower radiopharmaceutical dose. However, considering the relationship between the dose and resolution for ^89^Zr-PET, further technical improvements are still required.

## Figures and Tables

**Table 1:** FDA-approved mAbs used for 89Zr-PET

Name	Trade Name	Type	Target
Atezolizumab	Tecentriq	humanized	PD-L1
Bevacizumab	Avastin	humanized	VEGF-A
Brentuximab vedotin	Adcentris	chimeric	CD30
Cetuximab	Erbitux	chimeric	EGFR
Daratumumab	Darzalex	human	CD38
Girentuximab	Rencarex	chimeric	Carbonic anhydrase-IX
Ibritumomab tiuxetan	Zevalin	mouse	CD20
Nimotuzumab	Theracim, Theraloc	humanized	EGFR
Nivolumab	Opdivo	human	PD-1
Obinutuzumab	Gazyva	humanized	CD20
Ofatumumab	Arzerra	human	CD20
Panitumumab	Vectibix	human	EGFR
Pembrolizumab	Keytruda	humanized	PD-1
Pertuzumab	Omnitarg	humanized	HER2
Rituximab	MabThera, Rituxan	chimeric	CD20
Tositumomab	Bexxar	mouse	CD20
Trastuzumab	Herceptin	humanized	HER2

## Abbreviations

PET	Positron emission tomography
FDG	^18^F-fluorodeoxyglucose
mAb	Monoclonal antibody
DFO	Desferrioxamine B
NP	Nanoparticle
FDA	Food and Drug Administration
HER2	Human epidermal growth factor receptor-2
EGFR	Epidermal growth factor receptor
VEGF-A	Vascular endothelial growth factor-A
CD	Cluster of differentiation
PSMA	Prostate specific membrane antigen
PD-L1	Programmed cell death ligand-1
PD-1	Programmed cell death protein-1
ADCs	Antibody–drug conjugates
T-DM1	Trastuzumab emtansine
SUV	Standardized uptake value
CD44v6	v6 splice variant of CD44
TAM	Tumor-associated macrophages
HDL	High-density lipoprotein
CAR	Chimeric antigen receptor
DBN	Desferrioxamine-N-chlorosuccinimide
hMSC	Human mesenchymal stem cells
